# Low Power Consumption Red Light-Emitting Diodes Based on Inorganic Perovskite Quantum Dots under an Alternating Current Driving Mode

**DOI:** 10.3390/nano8120974

**Published:** 2018-11-26

**Authors:** Jingjing Liu, Zhangbo Lu, Xianju Zhang, Yangyi Zhang, Haiguang Ma, Yang Ji, Xiangxing Xu, Linwei Yu, Jun Xu, Kunji Chen

**Affiliations:** 1 National Laboratory of Solid State Microstructures, School of Electronic Science and Engineering and Collaborative Innovation Center of Advanced Microstructures, Nanjing University, Nanjing 210093, China; ljjhenan@163.com (J.L.); luzhangbo123@outlook.com (Z.L.); yyzhan@hotmail.com (Y.Z.); mhgnju@gmail.com (H.M.); Jiyang2018@hotmail.com (Y.J.); yulinwei@nju.edu.cn (L.Y.); kjchen@nju.edu.cn (K.C.); 2 Jiangsu Provincial Key Laboratory of Advanced Photonic and Electronic Materials, Nanjing University, Nanjing 210093, China; 3 Jiangsu Key Laboratory of Biofunctional Materials School of Chemistry and Materials Science, Nanjing Normal University, Nanjing 210046, China; Xianjuzhang2018@hotmail.com (X.Z.); xuxx@nju.edu.cn (X.X.)

**Keywords:** perovskite quantum dots, light emitting diodes, alternating current driving, low power consumption, silicon

## Abstract

Inorganic perovskites have emerged as a promising candidate for light-emitting devices due to their high stability and tunable band gap. However, the power consumption and brightness have always been an issue for perovskite light-emitting diodes (PeLEDs). Here, we improved the luminescence intensity and decreased the current density of the PeLEDs based on CsPbI_3_ quantum dots (QDs) and p-type Si substrate through an alternating current (AC) driving mode. For the different driving voltage modes (under a sine pulsed bias or square pulsed bias), a frequency-dependent electroluminescent (EL) behavior was observed. The devices under a square pulsed bias present a stronger EL intensity under the same voltage due to less thermal degradation at the interface. The red PeLEDs under a square pulsed bias driving demonstrate that the EL intensity drop-off phenomenon was further improved, and the integrated EL intensity shows the almost linear increase with the increasing driving voltage above 8.5 V. Additionally, compared to the direct current (DC) driving mode, the red PeLEDs under the AC condition exhibit higher operating stability, which is mainly due to the reducing accumulated charges in the devices. Our work provides an effective approach for obtaining strong brightness, low power consumption, and high stability light-emitting devices, which will exert a profound influence on coupling LEDs with household power supplies directly.

## 1. Introduction

Perovskite materials are emerging as semiconductors with potential applications in optoelectronic devices [1,2,3,4,5,6,7,8]. Their characteristics of high color purity [9,10,11,12], tunable band gap [13,14,15,16,17], and low cost [18,19] are favorable for obtaining light-emitting diodes (LEDs). However, the poor operating stability of perovskite light-emitting diodes (PeLEDs) directly obstructs commercial applications, especially for red light emitting devices. For the perovskite materials, the air-exposure stability of the inorganic perovskite (CsPbX_3_, X = Cl, Br, I) is superior to organic perovskite (CH_3_NH_3_PbX_3_, X = Cl, Br, I) as a result of replacing methyl ammonium with Cs cations [20,21,22]. This is crucial for practical optoelectronic applications, where long-term stability and cost effectiveness are vital. In previous works [23,24,25,26], the PeLEDs were usually studied under a direct current (DC) driving condition and they have one thing in common: the electroluminescent (EL) intensity drops off obviously at a high driving voltage due to heat generation and charge accumulation in the defect states, which would increase the power consumption of the devices. As a consequence, it is necessary to suppress heat generation and reduce charge accumulation in the defect states to obtain low power consumption devices.

EL devices operating under alternating current (AC) electricity have attracted more and more attention: one reason is the unique light-emitting mechanism of carrier generation and recombination; the other important one is their great potential application in sensors, lighting, and displays [27,28,29,30]. The AC condition has the advantage of reversal voltage, avoiding more charge accumulation at the device’s interface and a short operating time resulting in less heat generation compared to DC mode. Moreover, the trapped charge can be extracted in each period resulting in less defect creation [31]. In our previous work, the device performance based on Si quantum dots (QDs)/SiO_2_ multilayers [31] and green PeLEDs [32] under the AC driving mode was studied, respectively. A high operating stability was obtained for the two different devices due to less thermal degradation of the device induced by less operation time.

In this work, an optimized multilayered structure of Al/p-Si/Poly-TPD/CsPbI_3_/ZnO/ITO was created as the PeLEDs. In order to improve luminescence intensity and reduce the power consumption, the luminescence and electrical properties of the devices under the sine pulsed bias and square pulsed bias driving modes, and DC driving mode, were studied systematically. For devices under different AC driving voltage modes (sine pulsed bias and square pulsed bias), frequency-dependent electroluminescent behavior was observed. The red PeLEDs under square pulsed bias driving demonstrates that the EL intensity drop-off phenomenon was further improved. The EL intensity was also further enhanced, and the current density was decreased at the same driving voltage. Our findings provide an effective approach for obtaining a strong brightness, low power consumption, and high stability light-emitting device, which will exert profound influence on directly coupled LEDs with household power supplies.

## 2. Experimental Details

We purchased PbI_2_, Cs_2_CO_3_, ZnCl_2_, oleylamine, and cyclohexane from Aladdln Industrial Corporation (Shanghai, China) and purchased 1-octadecene and oleic acid from Alfa Aesar (Shanghai, China). The experimental details of synthesis of CsPbI_3_ QDs can be found in our previous work [32,33]. Direct centrifugation was used to separate the CsPbI_3_ QDs from the reaction solution. The precipitated CsPbI_3_ QDs were re-dispersed in cyclohexane.

The LEDs were fabricated on p-type Si wafers. The Si wafer was firstly cleaned with a mixture of hydrogen peroxide and ammonium hydroxide, then cleaned with a mixture of hydrogen peroxide and hydrochloride acid. Lastly, the Si wafer was cleaned with deionized water and dried by nitrogen. An aluminum electrode was deposited by thermal evaporation. The concentration of poly-TPD was 4 mg mL^−1^ and was spin-coated onto the p-Si substrate at 4000 rpm for 60 s. The concentration of CsPbI_3_ QDs was 10 mg mL^−1^ and was spin-coated onto the poly-TPD layer at 2000 rpm for 60 s. ZnO was deposited using a magnetron sputtering system. The ITO electrode was a circle with a diameter of 1.5 mm, which was deposited by the magnetron sputtering system through a circle shadow mask.

The microstructures of CsPbI_3_ QDs were characterized by transmission electron microscopy (TEM, TecnaiG2F20, Hillsboro, OR, USA) and the structure of CsPbI_3_ QDs were characterized by X-ray powder diffractometer (XRD, Rigaku, Akishima, Tokyo, Japan). Field emission scanning electron microscopy (FESEM, Sigma, Oberkochen, Germany) was employed to characterize the cross-sectional morphologies of the device. Optical absorption spectra of CsPbI_3_ QDs were obtained through a Ultraviolet-Visible-Near Infrared (UV-VIS-NIR) spectrometer (UV 3600, Shimadzu, Kyoto, Japan). A HORIBA Jobin Yvon system with a synapse (photomultiplier tube) PMT detector was utilized to obtain the photoluminescence spectra and the EL signals under both AC and DC bias. All the tests were conducted under room temperature.

## 3. Results and Discussion

**Figure 1:**
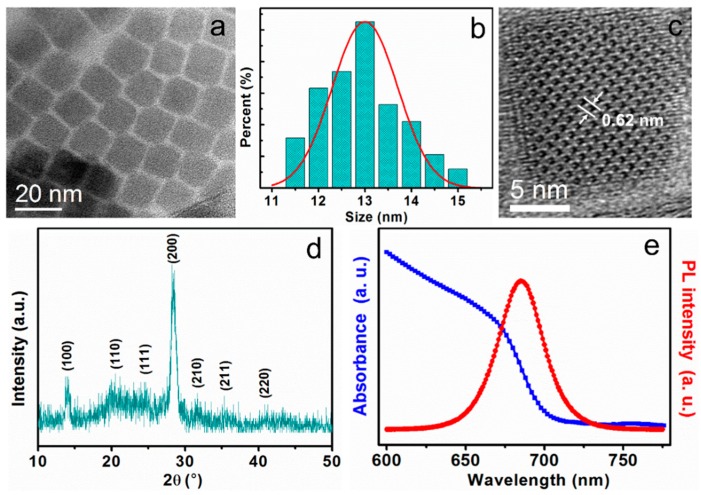
(**a**) TEM image of monodisperse CsPbI_3_ quantum dots (QDs); (**b**) Histogram for the size distribution of the CsPbI_3_ QDs; (**c**) High-resolution TEM (HRTEM) image of monodisperse CsPbI_3_ QDs; (**d**) The XRD spectrum of CsPbI_3_ QDs; (**e**) UV-vis absorption and photoluminescence (PL) spectra (with 325 nm excitation wavelength) of CsPbI_3_ QDs.

Figure 1a presents the transmission electron microscopy (TEM) image of CsPbI_3_ QDs, in which the monodisperse size distribution of QDs can be identified. The histogram of the size distribution of the CsPbI_3_ QDs demonstrates that CsPbI_3_ QDs have an average diameter of ~13 nm, and the size distribution range from 11 to 15 nm are shown in Figure 1b. In the high-resolution TEM (HRTEM) image shown in Figure 1c, the crystal lattice is clearly resolved with the lattice spacing of 0.62 nm, which corresponds to the (100) crystallographic plane of CsPbI_3_ [11,34]. The XRD pattern of CsPbI_3_ QDs is displayed in Figure 1d. The characteristic diffraction peaks at 13.92°, 20.17°, 24.53°, 28.33°, 31.67°, 35.27°, and 40.82° can be assigned to the diffractions from (100), (110), (111), (200), (210), (211), and (220) crystal planes, respectively. These results demonstrate that inorganic perovskite quantum dots (IPQDs) can be attested to the bulk cubic CsPbI_3_ with high crystalline quality. Figure 1e shows the absorption and photoluminescence (PL) spectra of CsPbI_3_ QDs. The absorption peak of CsPbI_3_ QDs is obvious and strong. Absorption onset occurs at ~702 nm for CsPbI_3_ QDs and a strong PL peak of CsPbI_3_ QDs is located at 685 nm. The full width at half maximum (FWHM) of the PL band is as narrow as 30 nm, which is helpful to obtain a high color purity.

**Figure 2:**
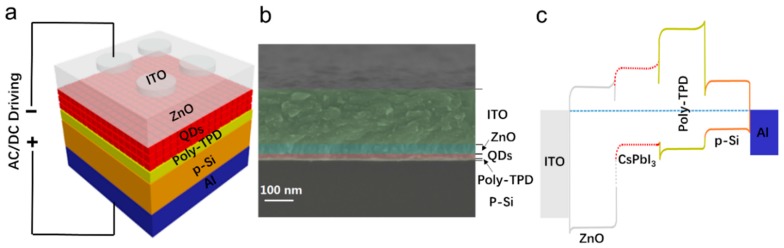
(**a**) Schematic structure of perovskite light-emitting diodes (PeLEDs); (**b**) SEM image of the cross section of PeLEDs; (**c**) Energy band diagrams of PeLEDs. AC: alternating current; DC: direct current.

PeLEDs were fabricated with a structure of ITO/ZnO/CsPbI_3_ QDs/poly-TPD/p-Si/Al (ITO: indium tin oxide, poly-TPD: poly [N, N′-bis (4-butylphenyl)-N, N′-bis (phenyl)-benzidine]), shown schematically in Figure 2a. Figure 2b presents cross-sectional SEM images of a complete PeLED. The thickness of the poly-TPD layer is about 10 nm and the CsPbI_3_ QDs layer is about 30 nm. The thickness of the ZnO and ITO film is about 45 and 230 nm, respectively. Figure 2c shows a schematic energy diagram of the device. Further, Al was used as anode, p-Si was used as a hole injection layer to decrease the hole barrier for efficient hole injection. Poly-TPD was used as hole transport and electron blocking layers, and CsPbI_3_ QDs were the emissive layer. ZnO was used as electron transport and hole blocking layers. ITO was utilized as a cathode. Light emission occurs when injected holes and electrons meet in the CsPbI_3_ QDs layer and recombine radiatively.

**Figure 3:**
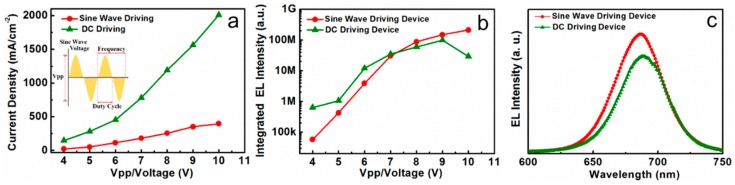
(**a**) Density-voltage curve of the CsPbI_3_ QDs light-emitting diodes (LEDs) under DC and AC (sine pulsed bias, duty cycle: 50%, frequency: 10 Hz) driving conditions. The inset is a schematic diagram of the sine pulsed bias; (**b**) Integrated electroluminescent (EL) intensity of CsPbI_3_ QDs LEDs versus voltage under DC and AC driving conditions (sine pulsed bias, duty cycle: 50%, frequency: 10 Hz); (**c**) The EL intensity of devices under DC (8 V) and AC (sine pulsed bias, duty cycle: 50%, frequency: 10 Hz, Vpp: 8 V) driving modes.

Figure 3a shows the current density-voltage (J-V) characteristics of the devices under AC and DC driving modes. The schematic diagram of the sine pulsed bias used in PeLEDs for AC driving is displayed in the inset of Figure 3a. The sine pulsed bias possesses the influence factors of Vpp, frequency, and duty cycle. For the sine pulsed mode, the driving Vpp ranges from 4 to 10 V, the duty cycle is 50%, and the excitation frequency is 10 Hz. When the device is driven under the DC mode, the driving voltage ranges from 4 to 10 V, which corresponds with AC driving Vpp. In previous work [33], the CsPbI_3_ QDs LEDs present current difference under the various bias polarizations due to the p-n heterojunction being formed in CsPbI_3_ QDs LEDs. As a result, the devices can only function under forward bias. For sine pulsed bias, the maximum forward voltage is equal to Vpp/2, as shown in the inset of Figure 3a. As exhibited in Figure 3a, the current density under AC bias is obviously lower than that of DC due to the working voltage under AC driving mode being lower than that under DC driving mode. Figure 3b shows the integrated EL intensity as a function of applied voltage under both the AC (sine pulsed bias) and DC driving conditions. The sine pulsed bias in Figure 3b presents 10 Hz frequency and 50% duty cycle. The integrated EL intensity under DC bias is greater than that under AC bias when the driving voltage is lower than 7 V. While under high driving voltage, the integrated EL intensity under DC bias becomes lower than that under AC bias. Furthermore, the integrated EL intensity shows a decreasing tendency when the DC driving voltage higher than 8.5 V. It is obvious that the EL intensity drop-off phenomenon under high voltage is greatly improved under the AC driving mode compared to the devices under the DC driving mode. Figure 3c shows the EL spectra of CsPbI_3_ QDs LEDs under DC (8 V) and AC driving modes (sine pulsed bias 8 V Vpp). It was found that the EL intensity under AC (sine pulsed bias) driving mode is improved by 26% than the DC driving one. Obviously, the devices working under an AC bias have the advantage of low power consumption. The improvement in light emission performance under AC drive mode can be attributed to suppressed heat generation at a high current density due to the short running time of the device.

**Figure 4:**
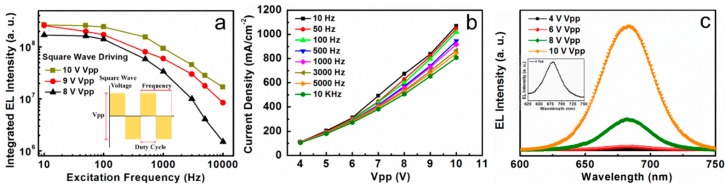
(**a**) The integrated EL intensity versus excitation frequency (from 10 Hz to 10 kHz) under different driving voltages with 50% duty cycle square pulsed bias. Illustration is a schematic diagram of square wave waveform; (**b**) Current density-voltage (J-V) characteristics of devices under different excitation frequency with 50% duty cycle square pulsed bias; (**c**) The EL intensity versus Vpp (from 4 to 10 V) with 10 Hz excitation frequency and 50% duty cycle under square pulsed bias. The inset shows the EL spectrum of the CsPbI_3_ QDs LEDs under 4 V Vpp.

In order to study device performance under different AC modes, we also apply the square pulsed bias on CsPbI_3_ QDs LEDs. Figure 4a shows the integrated EL intensity against the excitation frequency (from 10 Hz to 10 kHz) under the square pulsed bias with 50% duty cycle and different driving voltage (Vpp from 8 to 10 V). With increasing driving voltage, the integrated EL intensity becomes stronger under the same excitation frequency. The light emission is frequency dependent in our devices and the integrated EL intensity from all devices decreases as the frequency increases. When the excitation frequency is lower than 100 Hz, integrated EL intensity changes gently. A stable and bright red-light emission is obtained when the CsPbI_3_ QDs LEDs are driven under a square pulsed bias with 8 V Vpp and 50 Hz excitation frequency. It demonstrates that AC driving LEDs can be used directly for household power supplies. The inset in Figure 4a is the schematic diagram of a square pulsed bias which possesses the same influence factors as a sine pulsed bias. Figure 4b is the J-V relationship of CsPbI_3_ QDs LEDs under the AC (square pulsed bias) driving condition. With increasing excitation frequency from 10 Hz to 10 kHz, the effective current density of CsPbI_3_ QDs LEDs decreased, which resulted in the integrated EL intensity decay. When the frequency rises above ∼10 kHz with increasing excitation frequency, more and more injected carriers will be electrically pumped out before they can recombine with the latter ones. Thus, the EL intensity and current density all drop with an increase of frequency. The CsPbI_3_ QDs LEDs show sharp EL peaks under various biases, as shown in Figure 4c, with peak wavelengths of 685 nm and a narrow FWHM of ∼30 nm. The inset in Figure 4c shows the EL spectrum of the CsPbI_3_ QDs LEDs under 4 V Vpp which also presents a strong luminescence peak.

**Figure 5:**
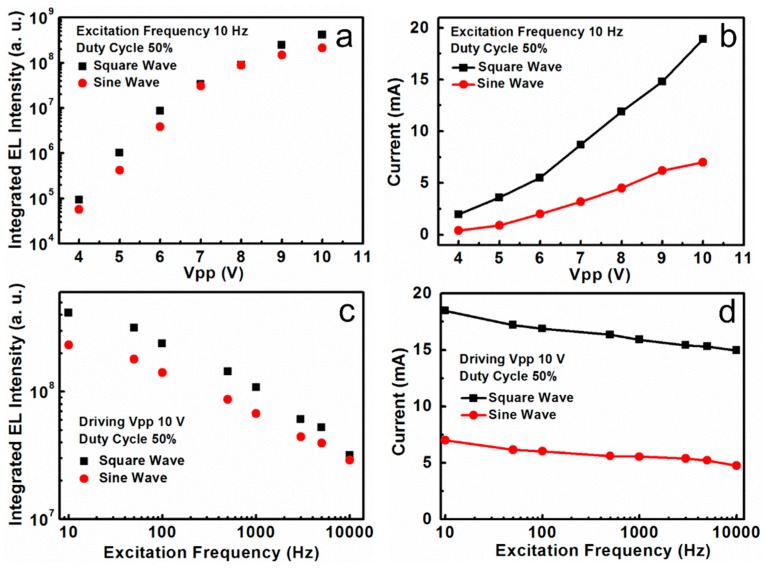
(**a**) The integrated EL intensity versus Vpp (from 4 to 10 V) with 10 Hz excitation frequency and 50% duty cycle under different driving voltage modes (square wave and sine wave); (**b**) Current versus Vpp for the devices under square pulsed bias and sine pulsed bias with 10 Hz excitation frequency and 50% duty cycle; (**c**) The integrated EL intensity versus excitation frequency (from 10 Hz to 10 kHz) with 10 V driving voltage and 50% duty cycle under different driving voltage modes (square pulsed bias and sine pulsed bias); (**d**) Current versus excitation frequency for the devices under square pulsed bias and sine pulsed bias with 10 V Vpp and 50% duty cycle.

The integrated EL intensity against the applied voltage under different driving modes (square pulsed bias and sine pulsed bias) is plotted in Figure 5a. The integrated EL intensity is gradually increased by increasing the driving voltage in all devices at a fixed frequency of 10 Hz and 50% duty cycle. For the different driving voltage modes (square pulsed bias and sine pulsed bias), the integrated EL intensity presents a similar changing trend. Furthermore, we noticed the integrated EL intensity under the square pulsed bias is stronger than that under the sine pulsed bias driving condition (Vpp from 4 to 10 V). When the driving Vpp is 10 V, the integrated EL intensity under the square pulsed bias is about twice as strong than that under the sine pulsed bias. There is often an emission intensity drop-off phenomenon of PeLEDs under high voltage for the DC driving mode [23,35]. The emission density under the AC driving mode obtains a significant improvement in the drop-off phenomenon. Figure 5b shows the working current density of the devices under different driving Vpp. For the two different driving voltage modes, the driving Vpp ranges from 4 to 10 V, the duty cycle and the excitation frequency is 50% and 10 Hz, respectively. Figure 5b exhibits high current flowing in the devices at a high driving voltage. As discussed above, the devices can only function under forward bias. As shown in the inset of Figure 4a, the working voltage of the square pulsed bias is equal to Vpp/2. Obviously, the working voltage under the square pulsed bias is higher than that under the sine pulsed bias at the same Vpp. As a result, the current under the square pulsed bias is higher than that under the sine pulsed bias, which would result in stronger emission intensity. The results are in good agreement with the observed results in Figure 5a. Figure 5c displays the influence of excitation frequency on the EL intensity. The excitation frequency ranges from 10 Hz to 10 kHz with a 10 V driving voltage and 50% duty cycle under a sine pulsed bias and a square pulsed bias. With increasing excitation frequency, all integrated EL intensity declined. The integrated EL intensity under the square pulsed bias is obviously stronger than that under the sine pulsed bias. As shown in Figure 5d, all currents are declining with an increasing excitation frequency from 10 Hz to 10 kHz, due to more and more injected carriers that will be electrically pumped out before they can recombine with the latter ones. The square pulsed current is a little higher than the sine pulsed current, because the working voltage under the square pulsed bias is higher than that under the sine pulsed bias at the same Vpp. The CsPbI_3_ QDs LEDs under the square pulsed bias present superior performance.

**Figure 6:**
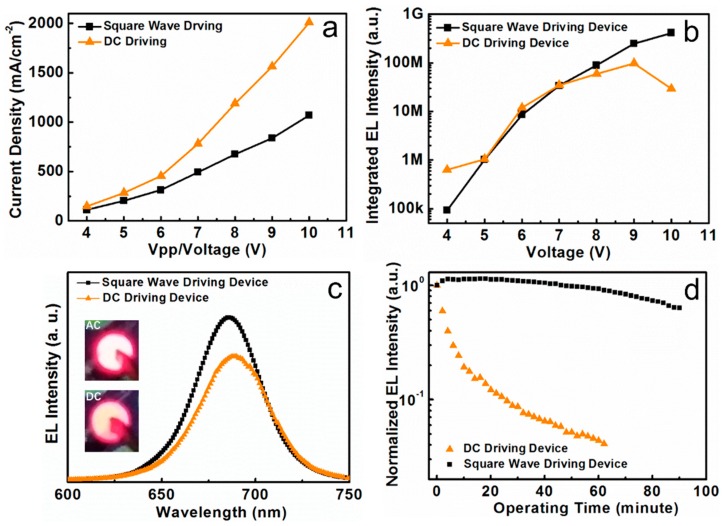
(**a**) Density-voltage curve of the CsPbI_3_ QDs LEDs under DC and AC (square pulsed bias, duty cycle: 50%, frequency: 10 Hz) driving conditions; (**b**) Integrated EL intensity of CsPbI_3_ QDs LEDs with different voltages under DC and AC driving conditions (square pulsed bias, duty cycle: 50%, frequency: 10 Hz); (**c**) The EL intensity of devices under DC (8 V) and AC (square pulsed bias, duty cycle: 50%, frequency: 10 Hz, Vpp: 8 V) driving conditions. The insets are the digital pictures showing the red-light emission from devices under AC and DC driving conditions; (**d**) Normalized emission decays of the PeLEDs after different running periods under DC (8 V) and AC (square pulsed bias, duty cycle: 50%, frequency: 10 Hz, Vpp: 8 V) driving conditions.

The effective current density under the square pulsed bias is obviously lower than that of the DC bias, as shown in Figure 6a, which demonstrates the devices working under AC bias have the advantage of low power consumption. The integrated EL intensities are plotted in Figure 6b as a function of applied bias under both the AC (square wave bias) and DC driving modes. The integrated EL intensity drops off after applying a DC bias higher than 8.5 V, but the integrated EL intensity under the square pulsed bias increases almost linearly with increasing driving voltage. The EL intensity drop-off phenomenon is further improved by square wave bias driving. Figure 6c shows the EL spectra of CsPbI_3_ QDs LEDs under DC and AC (square wave bias) driving modes (8 V bias). All the peaks of the devices were equipped with 30 nm FWHM. It was noticed that the EL intensity under the square wave bias was improved by 33% than that under DC driving. The insets are the digital pictures showing the red-light emission from devices under AC and DC driving conditions. In our previous work [33], the brightness of the CsPbI_3_ QDs LEDs under the DC driving condition reach the maximum value of 665 cd m^−2^. The brightness of the CsPbI_3_ QDs LEDs under the AC driving condition is about 850 cd m^−2^ at 8 V, which is estimated according to the integrated EL intensity. The enhanced luminescence performance under the AC driving condition can be attributed to the suppressed charge accumulation. In view of CsPbI_3_ QDs LEDs only working under a positive voltage bias because of the existing p-n heterojunction [33], the negative voltage bias of the AC mode will extract the trapped carriers, and then avoid more charge accumulation than that of the DC mode in the device.

In order to better understand the effect of driving modes on the PeLEDs, long-term operation tests in an ambient environment was further performed. The operation stability of devices under DC bias and AC (square pulsed) bias is shown in Figure 6d. We obtained the EL intensity decay variation of unpackaged devices in an atmospheric environment. During the test, a continuous 8 V/8 V Vpp voltage is injected and the EL intensity is recorded every two minutes. Under the DC driving mode, there is a 96% emission decay after 60 min. However, there is only a 36% emission decay after 90 min of operating time under the AC driving mode and the stability of the device is improved significantly. The operational stability under the AC driving mode is superior to PeLEDs under the DC driving condition, which can be attributed to less thermal degradation induced by the shorter operation time under the frequent reverse of applied bias. Also, the current density was smaller than that under the DC driving mode with the same applied voltage, which is also helpful for reducing power consumption and improving the device endurance.

## 4. Conclusions

We designed and fabricated LEDs that combined the inorganic perovskite CsPbI_3_ QDs with the p-type Si substrate. The luminescence and electrical properties of the devices under the AC driving mode and the DC driving mode were studied systematically. The frequency-dependent EL behavior was observed. With increasing excitation frequency, the emission density decreased. For the different AC driving voltage modes (square pulsed bias and sine pulsed bias), the integrated EL intensity of devices under the square pulsed bias was stronger than that under the sine pulsed bias due to the higher injection current. The resulting red PeLEDs under AC driving demonstrate a relatively low power consumption, and the EL intensity drop-off phenomenon is obviously improved compared to that under DC driving. The decay of emission intensity is less than 36% after 90 min for the AC driving condition, which demonstrates that the PeLEDs exhibit higher operating stability compared to the DC driving one due to the reduced accumulated charges in the devices. It is greatly significant to study the luminescence and electrical properties under the AC driving mode by applying different mode pulsed biases. The findings will exert a profound influence on directly coupled LEDs with household power supplies.
